# Cerebral Amyloid and Hypertension are Independently Associated with White Matter Lesions in Elderly

**DOI:** 10.3389/fnagi.2015.00221

**Published:** 2015-12-01

**Authors:** Julia A. Scott, Meredith N. Braskie, Duygu Tosun, Paul M. Thompson, Michael Weiner, Charles DeCarli, Owen T. Carmichael, the Alzheimer’s Disease Neuroimaging Initiative

**Affiliations:** ^1^IDeA Laboratory, Department of Neurology, University of California, DavisDavis, CA, USA; ^2^Imaging Genetics Center, Keck School of Medicine, University of Southern CaliforniaMarina del Rey, CA, USA; ^3^Center for Imaging Neurodegenerative Diseases, VA Medical Center, University of California, San FranciscoSan Francisco, CA, USA; ^4^Brain and Metabolism Imaging in Chronic Disease Lab, Pennington Biomedical Research Center, Louisiana State UniversityBaton Rouge, LA, USA

**Keywords:** hypertension, amyloid, FLAIR, MRI, normal aging, ADNI

## Abstract

In cognitively normal (CN) elderly individuals, white matter hyperintensities (WMH) are commonly viewed as a marker of cerebral small vessel disease (SVD). SVD is due to exposure to systemic vascular injury processes associated with highly prevalent vascular risk factors (VRFs) such as hypertension, high cholesterol, and diabetes. However, cerebral amyloid accumulation is also prevalent in this population and is associated with WMH accrual. Therefore, we examined the independent associations of amyloid burden and VRFs with WMH burden in CN elderly individuals with low to moderate vascular risk. Participants (*n* = 150) in the Alzheimer’s Disease Neuroimaging Initiative (ADNI) received fluid attenuated inversion recovery (FLAIR) MRI at study entry. Total WMH volume was calculated from FLAIR images co-registered with structural MRI. Amyloid burden was determined by cerebrospinal fluid Aβ_1-42_ levels. Clinical histories of VRFs, as well as current measurements of vascular status, were recorded during a baseline clinical evaluation. We tested ridge regression models for independent associations and interactions of elevated blood pressure (BP) and amyloid to total WMH volume. We found that greater amyloid burden and a clinical history of hypertension were independently associated with greater WMH volume. In addition, elevated BP modified the association between amyloid and WMH, such that those with either current or past evidence of elevated BP had greater WMH volumes at a given burden of amyloid. These findings are consistent with the hypothesis that cerebral amyloid accumulation and VRFs are independently associated with clinically latent white matter damage represented by WMHs. The potential contribution of amyloid to WMHs should be further explored, even among elderly individuals without cognitive impairment and with limited VRF exposure.

## Introduction

White matter hyperintensities (WMH) on fluid attenuation inversion recovery (FLAIR) magnetic resonance imaging (MRI) scans of the brain are commonly observed in elderly individuals, even in those without cognitive impairment (Yoshita et al., 2006; Brickman, 2013). Epidemiological studies have identified greater burden of WMHs as a risk factor for a variety of adverse outcomes, including dementia. Biological pathways leading to WMHs, and prevention or amelioration of WMH burden are therefore of major scientific interest.

Evidence suggests that several brain injury processes, including chronic hypoperfusion of tissues, impaired autoregulation of cerebral blood flow, venous collagenosis, and reactive gliosis contribute to WMHs in the elderly (Black et al., 2009; Gouw et al., 2011). Further evidence suggests that systemic vascular processes including arterial stiffness, atherosclerotic plaque deposition, and chronic inflammation contribute to these brain injury processes (Gouw et al., 2011). A substantial body of epidemiology research supports the pathway from systemic vascular factors to WMHs by identifying associations between indicators of systemic vascular injury (including prevalent clinically identified vascular risk factors (VRFs), clinically diagnosed vascular diseases, and biomarkers of vascular status) and presence of WMHs both contemporaneously and in the future (Bots et al., 1993; Swan et al., 1998; Romero et al., 2009; Debette et al., 2011). Basic science research further supports this association (Cognat et al., 2014). Following this evidence, reduction in systemic vascular injury (e.g., reduction in prevalence of VRFs) is frequently proposed as a means for preventing or lessening WMH accrual and thus reducing risks of adverse late life outcomes (Launer et al., 2010; Godin et al., 2011; Baumgart et al., 2015).

However, prior epidemiological studies of the association between systemic vascular injury and WMHs largely did not measure cerebral amyloid, which is known to be highly prevalent among elderly individuals, including those lacking clinically significant cognitive impairment (Price and Morris, 1999; Pike et al., 2007). Amyloid accumulation within blood vessels (i.e., cerebral amyloid angiopathy, CAA) could exacerbate cerebrovascular injury processes that contribute to WMHs; cerebrovascular dysfunction associated with systemic vascular injury could conversely exacerbate amyloid accrual by disrupting amyloid clearance via perivascular spaces (Preston et al., 2003; Snyder et al., 2015). In addition, amyloid deposition could contribute to WMHs by promoting injury processes that are not necessarily vascular in nature, including neuroinflammation, oxidative stress, and reactive oxygen species production (Yamada and Naiki, 2012; Snyder et al., 2015). To date, however, it is unclear which (if any) of these hypothetical scenarios commonly occurs in elderly individuals, and in particular there has been very little study of the association between cerebral amyloid and WMHs independent of systemic vascular injury processes (Brickman et al., 2015). Understanding whether cerebral amyloid is associated with WMH independent of systemic vascular processes is critically important to clarify the viability of proposed efforts to prevent WMHs through prevention of systemic vascular injury (Launer et al., 2010; Godin et al., 2011; Baumgart et al., 2015). In particular, an independent association between amyloid and WMH would leave open the possibility that amyloid accumulation could foil such WMH prevention efforts by promoting WMHs on its own.

This study investigates whether amyloid burden and VRFs are independently associated with WMH burden in cognitively normal (CN) elderly individuals. Using data from the Alzheimer’s Disease Neuroimaging Initiative (ADNI), which includes individuals with low to moderate levels of VRF exposure, we used FLAIR to measure WMHs, positron emission tomography (PET) and cerebrospinal fluid (CSF) assays to measure cerebral amyloid burden, and clinical histories of VRF exposure and conventional clinical measurements to characterize systemic vascular injury. We used this data to assess amyloid and systemic vascular indicators as independent predictors of WMH burden.

## Materials and Methods

### Participants

Data used in the preparation of this article were obtained from the ADNI database^1^. The ADNI was launched in 2003 by the National Institute on Aging (NIA), the National Institute of Biomedical Imaging and Bioengineering (NIBIB), the Food and Drug Administration (FDA), private pharmaceutical companies and non-profit organizations, as a $60 million, 5-year public–private partnership. The primary goal of ADNI has been to test whether serial MRI, PET, other biological markers, and clinical and neuropsychological assessment can be combined to measure the progression of mild cognitive impairment (MCI) and early Alzheimer’s disease (AD). Determination of sensitive and specific markers of very early AD progression is intended to aid researchers and clinicians to develop new treatments and monitor their effectiveness, as well as lessen the time and cost of clinical trials.

The Principal Investigator of this initiative is Michael W. Weiner, MD, VA Medical Center and University of California at San Francisco. ADNI is the result of efforts of many co-investigators from a broad range of academic institutions and private corporations, and subjects have been recruited from over 50 sites across the US and Canada. The initial goal of ADNI was to recruit 800 subjects but ADNI has been followed by ADNI-GO and ADNI-2. To date these three protocols have recruited over 1500 adults, ages 55 to 90, to participate in the research, consisting of cognitively normal older individuals, people with early or late MCI, and people with early AD. The follow up duration of each group is specified in the protocols for ADNI-1, ADNI-2, and ADNI-GO. Subjects originally recruited for ADNI-1 and ADNI-GO had the option to be followed in ADNI-2. For up-to-date information^2^.

The principles of informed consent in the current edition of the Declaration of Helsinki and applicable HIPAA privacy notifications was implemented before protocol procedures were carried out. Informed consent was obtained in accordance with US 21 CFR 50.25. Information was given in both oral and written form to subjects, their relatives, guardians or authorized representatives and study partners as deemed appropriate by the sites’ IRB. The consent form generated by the investigator with the assistance of the ADNI coordinating committee (ADNI-CC) was approved, along with the protocol, and HIPAA privacy notifications by the IRB and were acceptable to the ADNI-CC. HIPAA privacy requirements were met by either inclusion of required HIPAA text within the IRB-approved consent document or by separate HIPAA research authorization, pursuant to local regulations.

An inclusion criterion of a modified Hachniski score of 4 or lower excluded people with probable dementia due to cerebrovascular disease. Elderly individuals were recruited from over 50 sites across the US and Canada across multiple phases (1, GO, 2). Subjects recruited for ADNI-1 and ADNI-GO had the option to be followed in ADNI-2. Data used here were obtained from 150 CN ADNI-GO and ADNI-2 participants. CN was determined by absence of memory complaint, normal daily functioning, normal memory function measured on the Wechsler Memory Scale, a Mini-Mental State Exam score between 24 and 30, and a Clinical Dementia Rating of 0. Of these, 150 participants had data for all variables analyzed. Group clinical and demographic data are reported in **Table 1**. Baseline data—i.e., data associated with the participant’s first FLAIR scan—were analyzed in this cross-sectional study.

**Table 1:** Study demographics, vascular parameters, cerebrospinal fluid (CSF) immunoassays, and imaging metrics.

	Cognitively normal (CN)
Participants	150
Sex (male)	51.3%
Age (yr)	73.7 (6.3)
Education (yr)	16.6 (2.5)
White–not Hispanic/Latino	89.3%
*APOE* e4 carriage	28.7%
White matter hyperintensity volume (ln)	1.18 (1.16)
Intracranial volume (ICV) (cm^3^)	1,486 (158)
PET AV45 (SUVR)	1.11 (0.18)
CSF A_1-42_ (pg/ml)	194.4 (50.4)
CSF t-tau (pg/ml)	65.9 (31.7)
CSF p-tau_181_ (pg/ml)	33.2 (15.9)
Current vascular status	
Body mass index (BMI)	27.2 (4.3)
Systolic blood pressure (mm Hg)	136 (16)
Diastolic blood pressure (mm Hg)	75 (10)
Fasting blood glucose (mg/dl)	99 (18)
Total serum cholesterol (mg/dl)	189 (37)
Vascular history index	
0	24.7%
1	40.7%
2	24.0%
3	10.0%
4	0.7%
Vascular history	
Hypertension^a^	44.0%
Dyslipidemia	14.0%
Diabetes mellitus	9.3%
Smoking	18.0%
MI/CVA/AF^b^	4.0%
Micro-hemorrhage	14.0%

*All numerical entries mean (standard deviation).*

*^a^Two participants had past, but not current hypertension.*

*^b^Myocardial Infarction/Stroke/Atrial Fibrillation*.

### MRI Protocol and WMH Measurement

Magnetic resonance imaging acquisition: 3D Axial T2-weighted FLAIR and T1-weighted MRI sequences were collected at each site. Exact protocols varied by scanner type. General FLAIR sequence characteristics were as follows: FoV read 280mm; FoV phase 100.0%; slice thickness 8.0 mm; TR 20 ms; TE 5 ms; flip angle, 40°. Standard 3D T1-weighted MPRAGE sequence characteristics were as follows: TE 4 ms; TR 9 ms; voxel dimensions 1.1 mm × 1.1 mm 1.2 mm.

The WMH measurement approach is detailed in Supplementary Materials. Briefly, non-brain tissues were removed from T1-weighted and FLAIR images, the two images were spatially aligned, and MRI field artifacts were removed. Images were warped to a standard template space. In a Bayesian approach, the likelihood of WMH was estimated from FLAIR signal characteristics, the prior probability of WMH occurrence was calculated from previous supervised segmentations of independent FLAIR images, and additional constraints were applied at every voxel.

Micro-hemorrhage (MCH) assessment methods may be obtained from the ADNI database (ADNI_Methods_MCH_20121213.pdf). T2*-weighted GRE images (TE, 20 ms; TR, 650 ms; flip angle, 20°; section thickness, 4 mm; section gap, 0 mm) were used to identify presence and location of MCH and other medical findings. In this study, we used the presence of definite lobar MCH as a categorical predictor of interest in our models. Lobar MCH is an imaging indicator of CAA; we used this predictor to test whether associations between amyloid and WMH were accounted for simply by the presence of CAA.

### PET Imaging and Analysis

Positron emission tomography scans were acquired using a standardized protocol, which can be obtained from the ADNI database (ADNIGO_PET_Tech_Manual_01142011.pdf). Detailed PET analytical methods can be obtained from the ADNI web site and prior publications (ADNI_AV45_Methods_JagustLab_04-29-14.pdf) (Landau et al., 2013). Briefly, averaged and smoothed florbetapir image data was coregistered to the corresponding T1 structural image to define cortical regions. In this study, we used florbetapir (AV45) cortical standard uptake volume ratio (SUVR) normalized by whole cerebellum as our summary measure of interest.

### CSF

Cerebrospinal fluid collection procedures can be obtained from the ADNI database and prior publications (ADNI_Methods_UPENN_Biomarker_20120710.pdf) (Shaw et al., 2011). The xMAP Luminex platform (flow cytometric method) and Innogenetics/Fujirebio AlzBio3 immunoassay kits (monoclonal assay) were used following procedures in place at the UPenn/ADNI Biomarker Laboratory, according to the kit manufacturer’s instructions and as described in previous publications (Shaw et al., 2011). These assays produced measurements for Aβ_1-42_, total tau, and p-tau_181_. In this study, we used total CSF Aβ_1-42_ as a variable of interest.

### Vascular History

We calculated a vascular history index (**Table 1**) by screening the database of participant medical histories (Provenzano et al., 2013). Each participant received one point for history of each of the following conditions: hypertension, dyslipidemia (high lipids or cholesterol), smoking, type II diabetes mellitus, atrial fibrillation, myocardial infarction, or stroke. The potential range is 0 to 7. As a consequence of screens with the Hachinski scale at study entry, the effective range in this study was 0 to 4. The vascular history index is redundant with the Hachinski scale for history of hypertension and stroke. Otherwise, the scales document different items. Vascular history index was used to determine the proportion of the sample that had multiple VRF (34.7%).

### Current Vascular Status

Current vascular status was determined clinically using conventional methods. We report body mass index (BMI), systolic and diastolic BP, total fasting serum cholesterol, and fasting blood glucose. Only systolic and diastolic BP, cholesterol, and glucose were used as independent variables of interest in statistical analyses.

### Statistical Analyses

The aim of this study was to model amyloid burden and VRF as predictors of WMH volume. Predictors of interest were CSF Aβ_1-42_, hypertension history, hyperlipidemia history, diabetes history, systolic BP, diastolic BP, pulse pressure, fasting serum cholesterol, fasting blood glucose, and MCH. Nuisance variables were age, intracranial volume (ICV), sex, years of education, and *APOE* e4 carrier status.

Several of these predictors were highly correlated with each other. We used linear ridge regression to produce valid parameter estimates in the presence of such correlations. Traditional linear regression is known to produce unstable parameter estimates in the presence of highly inter-correlated variables, thus leading to regression findings that fail to generalize well to new data sets (Belsley et al., 1980; van der Kooij, 2007). Ridge regression reduces this instability by pushing regression parameter estimates closer to zero, thus resulting in more conservative parameter estimates. In addition, this conservative parameter biasing obviates the need for multiple comparison correction (van der Kooij, 2007). All predictors and the WMH outcome variable in ridge regression models were standardized by subtracting the mean and dividing by the standard deviation. Thus the ridge regression model did not require an intercept term. Missing data was excluded listwise, such that an individual was excluded from the model if it lacked data for any of the predictors. The model was resampled 250 times by bootstrapping to ensure stable parameter estimates.

In a separate ridge regression model, we determined whether the association between cerebral amyloid and WMH volume differed according to exposure to elevated BP. To do so, we categorized subjects into current systolic BP status groups according to whether their current systolic BP was below or above 140 mm Hg. We then categorized subjects into two BP exposure groups: (1) normal current systolic BP and negative history of hypertension and (2) either high current systolic BP or positive history of hypertension, or both. Then a ridge regression model was run with WMH volume as the outcome variable, and age, ICV, hypertension history, current BP status, CSF amyloid, BP exposure group, and BP exposure by CSF amyloid interaction.

All statistical models were estimated in SPSS v.21 with the CATREG algorithm. These analyses were replicated with PET AV45, in place of CSF Aβ_1-42,_ as the measurement of cerebral amyloid burden. Results of these models are reported in Supplementary Materials.

## Results

The characteristics of this sample of 150 CN participants are similar to other ADNI study samples (**Table 1**). Mean CSF Aβ_1-42_ and PET AV45 were close to the associated thresholds for positive categorization (192 pg/ml and 1.11 SUVR, respectively); thus a portion of this CN group showed evidence of cerebral amyloidosis. This group had low to moderate vascular risk based on vascular history index and only one subject had a VRF score of 4. The most common VRF was hypertension. On average, current vascular status indicators were near threshold for abnormally high (systolic BP, glucose, and cholesterol).

### Amyloid Burden and Hypertension are Associated with WMH Volume

In CN, greater age (β = 0.127, *p* = 0.001) and ICV (β = 0.114, *p* = 0.002), lesser Aβ_1-42_ (β = -0.135, *p* < 0.001), and positive hypertension history (β = 0.074, *p* = 0.032) were associated with greater WMH volume (adjusted *R*^2^ = 0.154, *p* = 0.013, see **Table 2**). Note that lesser Aβ_1-42_ indicates greater cerebral amyloid burden. All other independent variables were not significant. Substituting PET AV45 for CSF Aβ_1-42_ resulted in a highly similar model with the same significant associations (Supplementary Material). Because hypertension was the only VRF associated with WMH volume in this model, subsequent analyses focused on associations between BP and WMH volume.

**Table 2:** White matter hyperintensity volume regression model for all independent variables.

	CN^a^		
	Beta	*p*	CC
Age (years)	0.127	**0.001**	**0.219**
ICV (cm^3^)	**0.114**	**0.002**	**0.254**
CSF A_1-42_ (pg/ml)	**– 0.135**	**0.0002**	**– 0.299**
Hypertension history		**0.017**	**0.134**
Negative	**– 0.066**		
Positive	**0.083**		
Hyperlipidemia history		0.626	-0.009
Negative	- 0.003		
Positive	0.020		
Diabetes history		0.609	0.028
Negative	- 0.0003		
Positive	0.003		
Micro-hemorrhage		0.633	0.045
Negative	- 0.004		
Positive	0.025		
Sex		0.817	- 0.073
Male	0.004		
Female	- 0.004		
Education (years)	- 0.039	0.290	- 0.071
*APOE* e4 carriage		0.174	0.129
Negative	0.027		
Positive	- 0.066		
Systolic blood pressure (mm Hg)	0.006	0.828	- 0.034
Diastolic blood pressure (mm Hg)	0.017	0.603	0.039
Pulse pressure (mm Hg)	0.007	0.826	0.026
Fasting serum total cholesterol (mg/dL)	0.020	0.561	0.085
Fasting serum glucose (mg/dL)	- 0.050	0.150	- 0.082

*^a^Parameter estimate, *p*-value, partial correlation; model statistics: adjusted *R*^2^ = 0.154, *F* = 2.113, *p* = 0.013. Bold values indicates significant after multiple comparisons correction*.

### Amyloid Burden Association with WMH Differs by Exposure to Elevated BP

**FIGURE 1:**
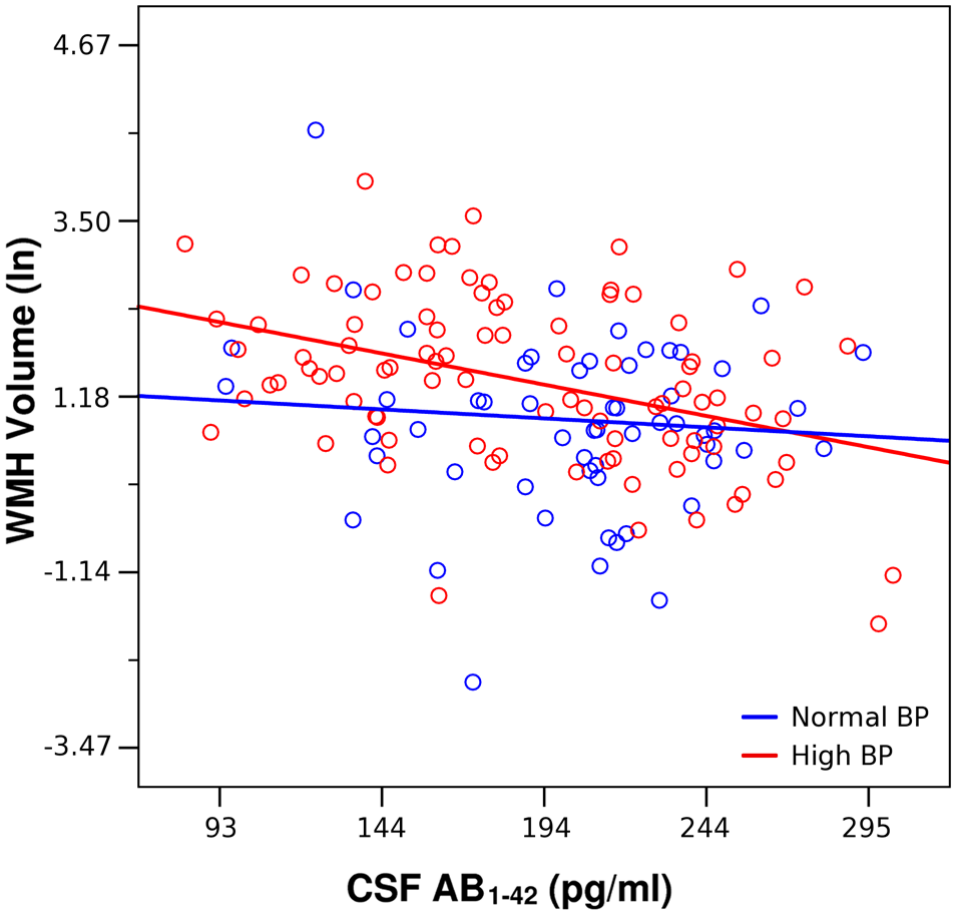
**Lesser CSF Aβ_1-42_ was associated with greater white matter hyperintensities (WMH) volume across all participants, adjusting for age and intracranial volume (**Table 2**)**. This association was driven by the high blood pressure (BP) group (red, β = –0.356, *p* < 0.001). CSF Aβ_1-42_ was not significantly associated with WMH volume in the Normal BP group (blue, β = – 0.102, *p* = 0.429).

In the second model, greater age, greater ICV, lesser CSF Aβ_1-42_, high BP exposure, and CSF Aβ_1-42_ by BP exposure group were significantly associated with greater WMH volume (**Table 3**). The BP exposure group significantly modified the association between CSF Aβ_1-42_ and WMH volume (**Table 3**; **Figure 1**). Specifically, among those who showed evidence of exposure to elevated BP, a low CSF Aβ_1-42_ (i.e., higher cerebral Aβ_1-42_) was associated with a greater WMH volume than in the normal BP group. Quantitatively, in the elevated BP group, each standard deviation decrease in CSF Aβ_1-42_ was associated with an 0.18-fold standard deviation increase in WMH; meanwhile in the normal BP group, each standard deviation decrease in CSF Aβ_1-42_ was only associated with an 0.01-fold standard deviation increase in WMH.

**Table 3:** White matter hyperintensity volume regression model with elevated blood pressure and amyloid interaction.

	CN^a^		
	Beta	*p*	CC
Age (years)	**0.109**	**0.003**	**0.212**
ICV (cm^3^)	**0.116**	**0.003**	**0.248**
Hypertension history		0.074	– 0.039
*Negative*	– 0.048		
*Positive*	0.061		
Systolic blood pressure (SBP)^b^		0.636	– 0.107
*Normal*	– 0.008		
*High*	0.013		
CSF A_1-42_ (pg/ml)	**– 0.086**	**0.001**	**– 0.019**
Blood pressure exposure group		**0.001**	**0.178**
*Normal*	**– 0.115**		
*High*	**0.070**		
CSF A_1-42_ by exposure group	**–0.097**	**0.0002**	**–0.097**

*^a^Parameter estimate, *p*-value, partial correlation; model statistics: adjusted *R*^2^ = 0.196, *F* = 5.244, *p* < 0.001.*

*^b^SBP at or above 140 mm Hg. Bold values indicates significant after multiple comparisons correction*.

**FIGURE 2:**
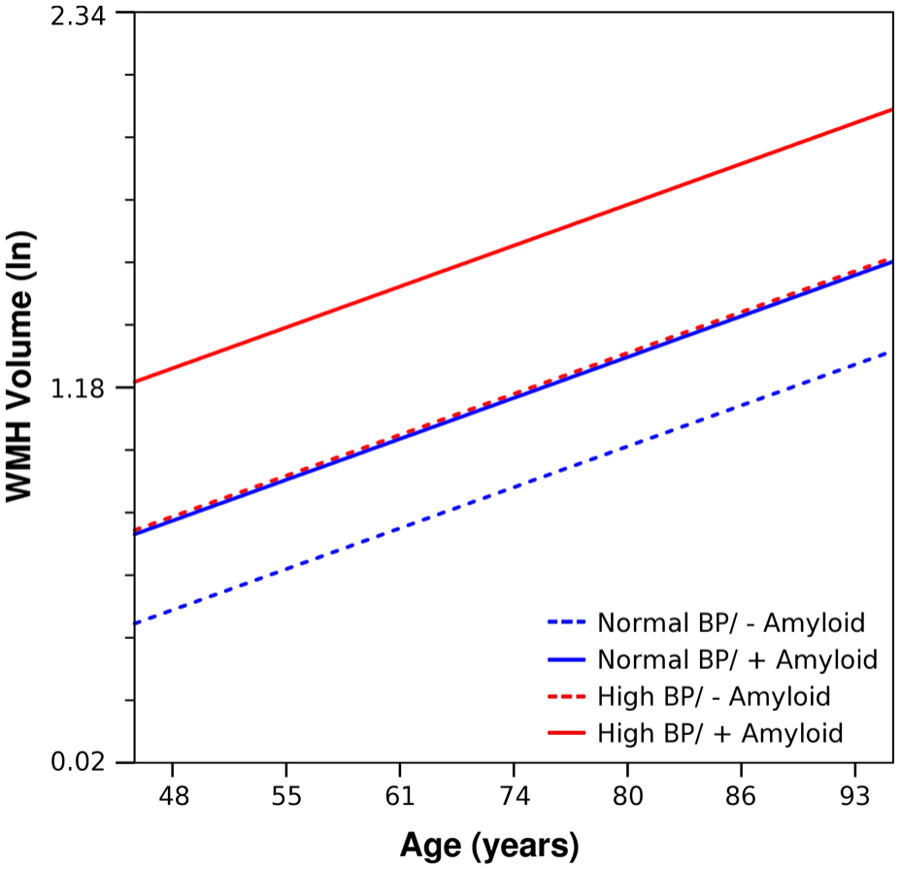
**Using parameter estimates from **Table 3**, estimated trends of WMH volume as a function of age, is shown for prototype individuals with mean ICV and normal BP/negative amyloid (blue, dashed), normal BP/positive amyloid (blue, solid), high BP/negative amyloid (red, dashed), and high BP/positive amyloid (red, solid)**. Amyloid positive threshold was CSF Aβ_1-42_ ≤ 192 pg/ml; the amyloid level used in these calculations is the mean for each category.

**Figure 2** gives an illustrative example of model-predicted WMH volumes for the population average ICV by age, exposure to elevated BP, and CSF Aβ_1-42_ burden. As expected, WMH volume is predicted to increase with age, and at each age WMH volume is predicted to be greater in those exposed to elevated BP. However, in individuals with high CSF Aβ_1-42_ burden and *no* evidence of elevated BP exposure, WMH burden is predicted to be about as high as in those exposed to elevated BP. Finally, the highest WMH burdens are predicted for those individuals who were both exposed to elevated BP and carry high CSF Aβ_1-42_ burden (**Table 4**).

**Table 4:** White matter hyperintensities (WMH) volume by CSF amyloid and systolic blood pressure/hypertension group.

	CSF amyloid		
	Negative	Positive^a^	
	
	WMH volume (ln)^**b**^	WMH volume (ln)^**b**^	Percent amyloid positive (total)
Normal blood pressure	0.74 (0.94)	0.95 (1.51)	33.3% (57)
High blood pressure	0.98 (1.07)	1.79(1.01)^c,d^	52.7% (93)

*^a^Amyloid positive defined by CSF Aβ_1-42_ < 192 pg/ml.*

*^b^Mean (standard deviation).*

*^c^Amyloid positive significantly greater than amyloid negative (*p* < 0.01).*

*^d^Significantly greater than low SBP and negative history of hypertension (*p**

## Discussion

There are several possible biological models for the relationships among amyloid accumulation, systemic vascular injury processes, and WMHs. In this study of cognitively healthy elderly individuals with low to moderate levels of vascular risk, we found that greater cerebral amyloid burden and exposure to elevated BP were independently associated with greater WMH volume, and that those with both elevated cerebral amyloid and exposure to elevated BP had greater WMH volume than could be accounted for by either of these factors in isolation. These results are consistent with multiple biological models: both amyloid and systemic vascular processes may be independently exacerbating WMH accrual; or systemic vascular processes may have accelerated both the deposition of amyloid and accrual of WMHs, leading to a downstream association between WMH and amyloid. Note that the presence of micro-hemorrhage was not a significant predictor of WMH, and thus we lack convincing evidence that CAA is the primary driver of associations between amyloid and WMH. The key implication of this finding is that both amyloidosis and prevalent VRFs should be considered as possible contributors to the loss of white matter health in cognitively normal individuals. More intense study of how the biological processes underlying systemic vascular injury and amyloid accrual independently or jointly contribute to WMH is needed to determine optimal strategies to arrest them and thus preserve white matter health late in life. For example, knowledge of these processes could clarify whether aggressive VRF control has the potential to preserve white matter health in the elderly or whether such an approach could be foiled by amyloidosis.

Of particular interest is the interaction between elevated BP and cerebral amyloid burden. Two earlier studies in non-demented elderly individuals have suggested that greater amyloid burden is associated with greater WMH burden (Stenset et al., 2006), or greater WMH accrual (Kaffashian et al., 2014), while adjusting for specific VRFs. Through path analysis, Stenset et al. (2006) demonstrated significant associations between hypertension and grade of WM lesion and then WM lesion and CSF amyloid. The recent Three-City Dijon study found that lower plasma amyloid was associated with greater WMH accrual, but did not find a significant interaction with hypertension or current BP (Kaffashian et al., 2014). To our knowledge, no other study to date has suggested that the relationship between cerebral amyloid and WMHs is modified by exposure to prevalent VRFs in cognitively normal elderly. If confirmed, this finding supports a mechanism in which long-term exposure to elevated BP and other vascular risks weakens the integrity of the cerebral vasculature, leaving it less well equipped to facilitate the clearance of amyloid as its production accelerates later in life (Thomas et al., 1996). In addition, the finding is consistent with a scenario in which systemic vascular injury processes and amyloid-related processes have a synergistic relationship, reinforcing each other to produce super-additive effects on white matter health (van Norden et al., 2012). Understanding these interactions will be important to understand how best to prevent the negative effects of these processes on white matter health.

Our ADNI-2 results mirror what was observed in ADNI-1 in a key way, in that VRFs and greater age were associated with greater WMH volume (Carmichael et al., 2010). That said, one prior ADNI-1 study did not find an association between WMH volume and CSF Aβ_1-42_, nor did it find an association between WMH volume and a vascular risk summary score in cognitively normal individuals (Lo et al., 2012). Methodological differences between the two studies may have driven these discrepancies. Although ADNI-1 collected measurements of amyloid, WMH, and vascular risk similar to ADNI-2, comparisons between the two studies are difficult for several reasons. First, past ADNI-1 studies modeled WMH as a predictor of various outcomes, while this study modeled WMH as the outcome (Lo et al., 2012; Barnes et al., 2013; Guzman et al., 2013; Haight et al., 2013; Nettiksimmons et al., 2013; Provenzano et al., 2013). Second, because amyloid measurement (via CSF) was optional in ADNI-1, the proportion of individuals in ADNI-1 with both WMH and amyloid measurements is far smaller than in ADNI-2. Third, WMH measurement techniques differed: ADNI-1 used less-sensitive PD- and T2-weighted imaging and 1.5T field strength, while ADNI-2 used more-sensitive FLAIR and 3T field strength (Wardlaw et al., 2013). In the study by Lo et al. (2012), the Framingham CVD Risk Score utilized in the prior ADNI-1 study includes age, gender, BMI, current BP, smoking history, and diabetes (D’Agostino et al., 2008). In our analysis, only age and positive history of hypertension, which is not part of CVD risk score, were associated with WMH volume, while current BP, smoking, diabetes, BMI, and gender did not. Further, the WMH volumes in the sample of ADNI-1 cognitively normal participants were on average much lower than those in the current ADNI-2 sample. These substantially lower WMH volumes may either be due to the lower sensitivity of imaging method or characteristic differences in the prevalence of white matter damage.

Our findings agree with those of prior studies outside of ADNI. History of hypertension was associated with greater WMH volume in our study, in agreement with findings from large epidemiological samples (Dufouil et al., 2005; Allan et al., 2014; Rosano et al., 2014). In contrast, current BP did not have an independent effect on WMH burden. Previous studies have also shown a more reliable association between WMH and hypertension history compared to that with current BP (de Leeuw et al., 1999; Verhaaren et al., 2013; Rosano et al., 2014). The hypothesis arising from this repeated finding is that the critical vascular determinant of WMH is the cumulative exposure to elevated BP over a prolonged period as reflected in clinical history of hypertension. Further, we showed that those with current or past hypertension had a stronger association between cerebral amyloid burden and total WMH volume.

The key strength of this study is that vascular factors, cerebral amyloid, and WMHs were measured in a large group of cognitively normal elderly individuals, using standardized methods. The key limitation of the study was that the depth of vascular ascertainment was shallow. ADNI does not actively recruit participants with high vascular risk burden, so we did not have the statistical power to detect effects of vascular risk as other studies with larger sampling of these conditions have demonstrated (Hughes et al., 2012; de Bruijn et al., 2014; Willey et al., 2014; Geijselaers et al., 2015). Advanced assays and more in-depth documentation of vascular history would provide a more precise characterization of current vascular status and may have provided a clearer picture of which specific processes may be involved in the development of WMHs. For example, comprehensive assessment of the lipid profile may elucidate a relationship between types of cholesterol and WMH that has been observed (Schmidt et al., 1996; de Bruijn et al., 2014; Willey et al., 2014). Since our study only included individuals whose burden of systemic vascular injury was low to moderate compared to typical epidemiological studies of aging, how relationships between cerebral amyloid and WMH extend to populations with a relatively high vascular injury burden needs to be studied.

## Conclusion

In conclusion, both amyloid burden and history of hypertension are independently associated with WMH burden in a cognitively normal subgroup of ADNI-2, a study with a relatively mild overall burden of vascular risk. Further, these two factors interacted, such that greater amyloid burden was associated with even more severe WMH accrual in the context of elevated BP exposure. Though the present analysis cannot determine the directionality of the relationship between WMH, cerebral amyloid, and VRFs, the significant associations emphasize the importance of better understanding how amyloid pathology and cardiovascular health affect brain health and function as we age.

## Author Contributions

JS had full access to all the data in the study and takes responsibility for the accuracy of the data analysis. Study concept and design: OC. Acquisition of data: AI. Analysis and interpretation of data: JS, CC, and OC. Drafting of manuscript: JS and OC. Critical revision of manuscript for important intellectual content: JS, MB, DT, MW, PT, CC, and OC. Statistical Analysis: JS DT, and OC. Obtained funding: CC and OC. Administrative, technical, and material support: CC, PT, and MW. Study supervision: OC.

## Conflict of Interest Statement

The authors declare that the research was conducted in the absence of any commercial or financial relationships that could be construed as a potential conflict of interest.

## Footnotes

1 www.adni.loni.usc.edu

2 www.adni-info.org
