# Supplementary material for: Cerebral Amyloid and Hypertension are Independently Associated with White Matter Lesions in Elderly
Source: Front Aging Neurosci. 2015 Dec 1;7:221. doi: 10.3389/fnagi.2015.00221 (PMC4664630; doi:10.3389/fnagi.2015.00221)
Supplement: Supplementary file 1 [file Table_1.PDF]

Supplementary Material

Cerebral amyloidosis and vascular risk factor history are independently associated with white matter hyperintensity in cognitively normal elderly individuals

Julia A. Scott*, Meredith N. Braskie, Duygu Tosun, Paul M. Thompson, Michael Weiner, Charles DeCarli, Owen T. Carmichael for Alzheimer's Disease Neuroimaging Initiative

Correspondence: Julia Scott: jascott@ucdavis.edu

Supplementary Tables

Ridge regression models for white matter hyperintensity (WMH) volume. Predictors of interest were PET AV45, hypertension history, hyperlipidemia history, diabetes history, systolic BP, diastolic BP, pulse pressure, fasting serum cholesterol, fasting blood glucose, and MCH. Nuisance variables were age, ICV, sex, years of education, and APOE e4 carrier status (Supplementary Table 1). In a separate ridge regression model, we determined whether the association between cerebral amyloid and WMH volume differed according to exposure to elevated blood pressure. WMH volume was the outcome variable, and age, ICV, hypertension history, current BP status, PET AV45, BP exposure group, and BP exposure by PET AV45 interaction were the independent variables (Supplementary Table 2).

Supplementary Table 1. White matter hyperintensity volume regression model for all independent variables with PET AV45

	**Cognitively Normal**[a]		
	Beta	p	CC
Age (years)	**0.119**	**0.002**	**0.206**
ICV (cm^3)	**0.115**	**0.002**	**0.231**
PET AV45 (SUVR)	**0.101**	**0.002**	**0.191**
Hypertension History		**0.018**	**0.161**
Negative	**-0.075**		
Positive	**0.096**		
Hyperlipidemia History		0.685	-0.007
Negative	-0.002		
Positive	0.012		
Diabetes History		0.814	0.011
Negative	-0.0003		
Positive	0.003		
Micro-hemorrhage		0.957	0.060
Negative	0.006		
Positive	-0.035		
Sex		0.601	-0.044
Male	0.076		
Female	-0.080		
Education (Years)	-0.039	0.264	-0.080
APOE e4 Carriage		0.277	0.087
Negative	0.033		
Positive	-0.082		
Systolic Blood Pressure (mm Hg)	0.025	0.399	0.027
Diastolic Blood Pressure (mm Hg)	0.015	0.640	-0.012
Pulse Pressure (mm Hg)	0.013	0.669	-0.022
Fasting Serum Total Cholesterol (mg/dL)	0.016	0.211	0.064
Fasting Serum Glucose (mg/dL)	-0.042	0.229	-0.067

[a] Parameter estimate, p-value, partial correlation; model statistics: n=150, adjusted R^2=0.116, F=1.766, p=0.046

Supplementary Table 2. White matter hyperintensity volume regression model with elevated blood pressure and amyloid (PET AV45) interaction

	**Cognitively Normal**[a]		
	Beta	p	CC
Age (years)	**0.114**	**0.004**	**0.230**
ICV (cm^3)	**0.114**	**0.004**	**0.238**
Hypertension History		0.073	-0.016
Negative	-0.050		
Positive	0.063		
Systolic Blood Pressure (SBP)[b]		0.442	-0.080
Normal	-0.014		
High	0.023		
PET AV45 (SUVR)	**0.070**	**0.003**	**0.106**
Blood Pressure Exposure Group		**0.001**	**0.152**
Normal	**-0.119**		
High	**0.073**		
PET AV45 by Exposure Group	**0.051**	**0.026**	**-0.060**

[a] Parameter estimate, p-value, partial correlation; model statistics: n=150, adjusted R^2=0.155, F=4.835, p<0.001

[b] Systolic blood pressure at or above 140 mm Hg
