# Supplementary material for: Cerebral Amyloid and Hypertension are Independently Associated with White Matter Lesions in Elderly
Source: Front Aging Neurosci. 2015 Dec 1;7:221. doi: 10.3389/fnagi.2015.00221 (PMC4664630; doi:10.3389/fnagi.2015.00221)
Supplement: Supplementary file 2 [file Presentation_1.PDF]

Supplementary Material

Cerebral amyloidosis and vascular risk factor history are independently associated with white matter hyperintensity in cognitively normal elderly individuals

Julia A. Scott*, Meredith N. Braskie, Duygu Tosun, Paul M. Thompson, Michael Weiner, Charles DeCarli, Owen T. Carmichael for Alzheimer's Disease Neuroimaging Initiative

Correspondence: Julia Scott: jascott@ucdavis.edu

1 Supplementary Data

Four Tissue Segmentation in ADNI II

Charles DeCarli, MD, Pauline Maillard, PhD, Evan Fletcher, PhD

Department of Neurology and Center for Neuroscience, University of California at Davis

Summary

While Alzheimer's disease (AD) is the most common cause for dementia among older individuals [1,2] and the primary goal of ADNI, the lifetime risk for stroke equals and may exceed the risk of AD in some circumstances [3]. In addition, MRI evidence of asymptomatic cerebrovascular disease (CVD) occurs in one-third of older individuals [4]. In community based studies, mixed pathologies account for the most common cause of dementia [5] and the impact of cerebrovascular and AD pathology is additive, particularly when the AD pathological burden is mild [6]. Similarly, MRI community based studies have shown that white matter hyperintensities (WMH) increase with advancing age and associated vascular risk factors [4,7] and are associated with an array of cognitive deficits in cross-sectional studies [8-10]. Similar cross-sectional differences are also seen with clinically silent brain infarctions noted on MRI [4,11,12]. Importantly, community based studies show that both WMH and SBI are associated with cognitive decline and *incident* MCI and dementia [13,14].

These results strongly suggest that vascular brain injury (WMH, SBI) occurs commonly amongst older community dwelling cognitively normal individuals and is associated with subtle cognitive impairment, including memory impairment. Importantly, increasing survival from vascular disease including hypertension, diabetes, myocardial infarction and stroke is likely to increase the prevalence of asymptomatic vascular brain disease thereby increasing the public health consequences of cognitive impairment related to these disorders. Furthermore, the age of onset of these diseases is quite early and cognitive effects appear to begin similarly early in life, possibly before age 60 [10,14,15].

Method

WMH Measurement

For ADNI 2, our WMH measurement approach is based a Bayesian approach to segmentation of high resolution 3D T1 and FLAIR sequences. In brief, non-brain structures are excluded from the 3D T1

images prior to measurement using an automated atlas based method. The FLAIR image is the affined transformed to the 3DT1 image using the FLIRT method from the FSL tool box, with error estimation based on correlation ratio. Inhomogeneity correction of the 3D T1is performed using interleaved bias estimation and B-Spline deformation with a template[16]. This multiple iteration method updates a B--spline intensity deformation between an unbiased template image and the subject image with an estimation of a bias field based on the current template--to--image alignment. The bias field is modeled using a spatially smooth thin--plate spline interpolation based on ratios of local image patch intensity means between the deformed template and subject images. FLAIR inhomogeneity corrections after co-registration of the FLAIR to the 3D T1 image is based on a previously published local histogram normalization method[17]. Prior to WMH calculation, each 3D T1 image is non-linearly aligned to a common template atlas and each of the accompanying images are transformed onto the same atlas using the same transformation parameters.

Estimation of WMH is then performed using a modified Bayesian probability structure based on a previously published method of histogram fitting [18]. Prior probability maps for WMH were created from more than 700 individuals with semi-automatic detection of WMH followed by manual editing. Likelihood estimates of the native image are calculated through histogram segmentation and thresholding. All segmentation is initially performed in standard space resulting in probability likelihood values of WMH at each voxel in the white matter (Figure 1). These probabilities are then thresholded at 3.5 sd above the mean to create a binary WMH mask. Segmentation is based on a modified Bayesian approach that combines image likelihood estimates, spatial priors and tissue class constraints. The segmented WMH masks are then back-transformed in to native space for tissue volume calculation. Since image segmentation occurs in a common template space, group statistics using previously reported methods[19-21] can be applied.

Gray, White and CSF Measurement

The primary segmentation mechanism is an Expectation-Maximization (EM) algorithm that iteratively refines its segmentation estimates to produce outputs that are most consistent with the input intensities from the native-space T1 images and a model of image smoothness [22,23]. Like all EM algorithms, the system must be initialized with a reasonable estimate. We produce this initial estimate from the template-space warps used for WMH detection; because locations of WM/GM/CSF tissues are known in the template space, transforming these masks back to the each image's native space produces rough estimate 3-tissue segmentations (Figure 2). We then calculate the mean and standard deviation of the image intensities in locations labeled as each tissue type, and in the main segmentation method these values form the initial parameters for a Gaussian model of image intensity for each class. At each iteration, the segmenter uses a Gaussian model of T1-weighted image intensity for each tissue class, in order to produce a segmentation. In the first iteration, these models are estimated as described above. The segmentation yielded by these appearance models alone is then refined using a Markov Random Field (MRF) model, a computational statistical method that efficiently produces a label map consistent with both the input intensities and image smoothness statistics. Inference in the MRF is computed using an adaptive priors model[23]. This refined segmentation from the MRF is then used to compute new Gaussian intensity models for each tissue class, and the algorithm repeats, iteratively switching between calculating Gaussian appearance models and MRF-based segmentation, until convergence. The MRF-based segmentation at the final iteration is used as the final output segmentation.

Final Four Tissue Segmentation

Final four tissue segmentation consists of substitution of voxels within the three tissue segmented image with voxels identified as WMH from the automatic WMH segmentation algorithm. Results are reported in native space as volumes in cubic centimeters. Total intracranial volume based on automatic segmentation is also included (Figure 2).

Robustness

Irregularities and variance in image intensities are corrected for in preprocessing by the best available methods. WMHs, a difficult problem for many similar segmenters, are detected using a dedicated specialized method to ensure their proper handling. While the method is sensitive to being given poor quality initial estimates, those we use are generated in a very sophisticated and robust automated fashion. The mathematical methods used are both state of the art and strongly validated.

About the Authors

This document was prepared by Charles DeCarli, University of California at Davis. For more information please contact Charles DeCarli at cdecarli@ucdavis.edu.

Bibliography

1. Sayetta RB. Rates of Senile Dementia, Alzheimer's Type, in the Baltimore Longitudinal Study. *J Chronic Dis.* 1986;39(4):271-286.
2. Evans DA, Funkenstein HH, Albert MS, et al. Prevalence of Alzheimer's Disease in a Community Population of Older Persons. Higher Than Previously Reported. *JAMA.* 1989;262(18):2551-2556.
3. Seshadri S, Beiser A, Kelly-Hayes M, et al. The Lifetime Risk of Stroke: Estimates from the Framingham Study. *Stroke.* 2006;37(2):345-350.
4. Decarli C, Massaro J, Harvey D, et al. Measures of Brain Morphology and Infarction in the Framingham Heart Study: Establishing What Is Normal. *Neurobiol Aging.* 2005;26(4):491-510.
5. Schneider JA, Arvanitakis Z, Bang W, Bennett DA. Mixed Brain Pathologies Account for Most Dementia Cases in Community-Dwelling Older Persons. *Neurology.* 2007;69(24):2197-2204.
6. Schneider JA, Wilson RS, Bienias JL, Evans DA, Bennett DA. Cerebral Infarctions and the Likelihood of Dementia from Alzheimer Disease Pathology. *Neurology.* 2004;62(7):1148-1155.
7. Jeerakathil T, Wolf PA, Beiser A, et al. Cerebral Microbleeds: Prevalence and Associations with Cardiovascular Risk Factors in the Framingham Study. *Stroke.* 2004;35(8):1831-1835.
8. DeCarli C, Murphy DG, Tranh M, et al. The Effect of White Matter Hyperintensity Volume on Brain Structure, Cognitive Performance, and Cerebral Metabolism of Glucose in 51 Healthy Adults. *Neurology.* 1995;45(11):2077-2084.
9. Swan GE, DeCarli C, Miller BL, et al. Association of Midlife Blood Pressure to Late-Life Cognitive Decline and Brain Morphology. *Neurology.* 1998;51(4):986-993.
10. Au R, Massaro JM, Wolf PA, et al. Association of White Matter Hyperintensity Volume with Decreased Cognitive Functioning: The Framingham Heart Study. *Arch Neurol.* 2006;63(2):246-250.
11. Das RR, Seshadri S, Beiser AS, et al. Prevalence and Correlates of Silent Cerebral Infarcts in the Framingham Offspring Study. *Stroke.* 2008;39(11):2929-2935.
12. Aggarwal NT, Wilson RS, Bienias JL, et al. The Association of Magnetic Resonance Imaging Measures with Cognitive Function in a Biracial Population Sample. *Arch Neurol.* 2010;67(4):475-482.
13. Debette S, Beiser A, DeCarli C, et al. Association of MRI Markers of Vascular Brain Injury with Incident Stroke, Mild Cognitive Impairment, Dementia, and Mortality. The Framingham Offspring Study. *Stroke.* 2010.
14. Debette S, Seshadri S, Beiser A, et al. Midlife Vascular Risk Factor Exposure Accelerates Structural Brain Aging and Cognitive Decline. *Neurology.* 2011;77(5):461-468.
15. Seshadri S, Wolf PA, Beiser A, et al. Stroke Risk Profile, Brain Volume, and Cognitive Function: The Framingham Offspring Study. *Neurology.* 2004;63(9):1591-1599.
16. Fletcher E, Carmichael O, DeCarli C. MRI Non-Uniformity Correction through Interleaved Bias Estimation and B-Spline Deformation with a Template. *Conference proceedings: Annual International Conference of the IEEE Engineering in Medicine and Biology Society. IEEE Engineering in Medicine and Biology Society. Conference.* 2012;2012:106-109.
17. DeCarli C, Murphy DG, Teichberg D, Campbell G, Sobering GS. Local Histogram Correction of Mri Spatially Dependent Image Pixel Intensity Nonuniformity. *Journal of Magnetic Resonance Imaging.* 1996;6(3):519-528.
18. DeCarli C, Miller BL, Swan GE, et al. Predictors of Brain Morphology for the Men of the Nhlbi Twin Study. *Stroke; A Journal of Cerebral Circulation.* 1999;30(3):529-536.

19. DeCarli C, Fletcher E, Ramey V, Harvey D, Jagust WJ. Anatomical Mapping of White Matter Hyperintensities (WMH): Exploring the Relationships between Periventricular WMH, Deep WMH, and Total WMH Burden. *Stroke; A Journal of Cerebral Circulation.* 2005;36(1):50-55.
20. Yoshita M, Fletcher E, Harvey D, et al. Extent and Distribution of White Matter Hyperintensities in Normal Aging, Mci, and Ad. *Neurology*. 2006;67(12):2192-2198.
21. Carmichael O, Schwarz C, Drucker D, et al. Longitudinal Changes in White Matter Disease and Cognition in the First Year of the Alzheimers Disease Neuroimaging Initiative. *Arch Neruol*. 2010; 67(11):1370-1378.
22. Rajapakse JC, Giedd JN, DeCarli C, et al. A Technique for Single-Channel Mr Brain Tissue Segmentation: Application to a Pediatric Sample. *Magnetic Resonance Imaging.* 1996;14(9):1053-1065.
23. Fletcher E, Singh B, Harvey D, Carmichael O, DeCarli C. Adaptive Image Segmentation for Robust Measurement of Longitudinal Brain Tissue Change. *Conference proceedings:Annual International Conference of the IEEE Engineering in Medicine and Biology Society. IEEE Engineering in Medicine and Biology Society. Conference.* 2012;2012:5319-5322.

2 Supplementary Figures and Tables

2.1 Supplementary Figures

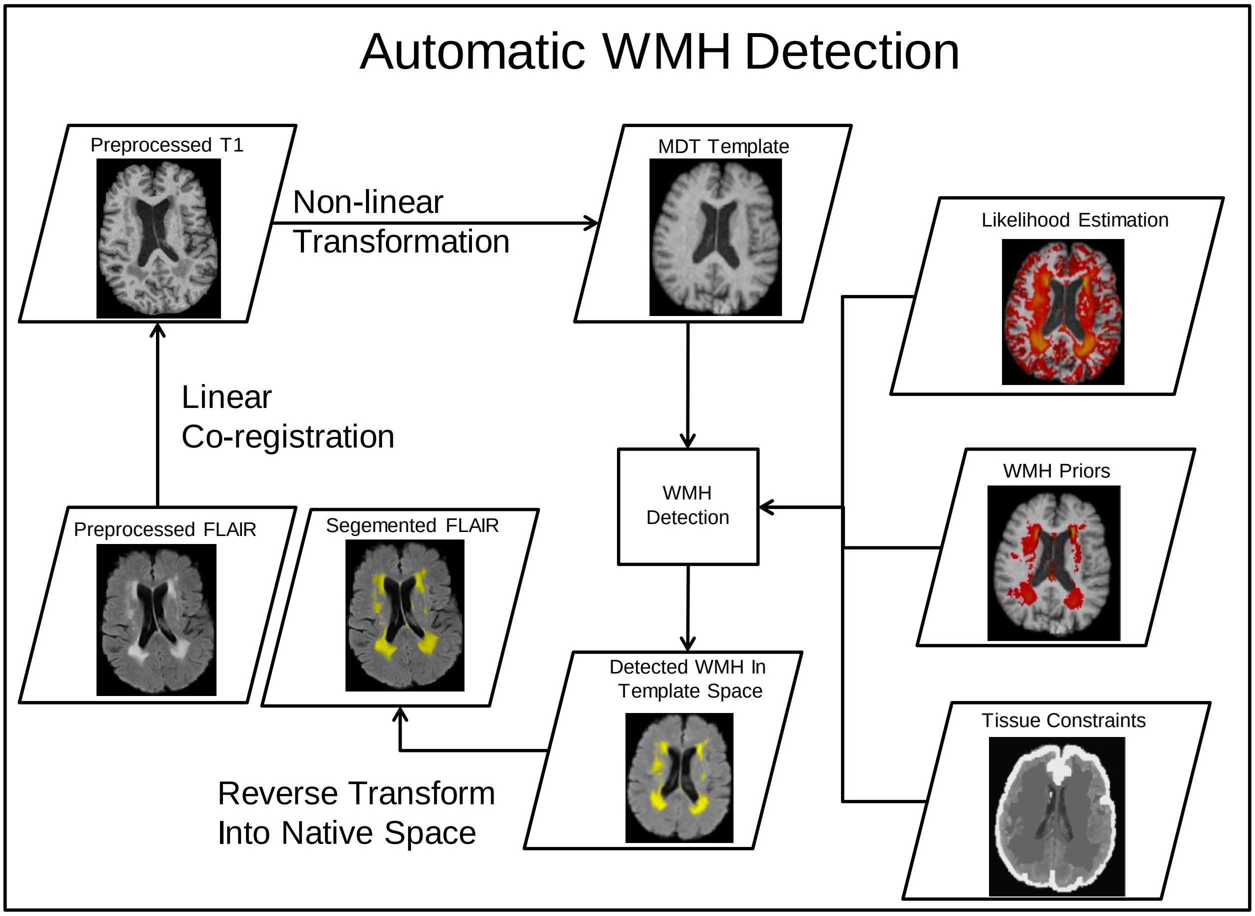

Supplementary Figure 1. WMH Segmentation Pipeline

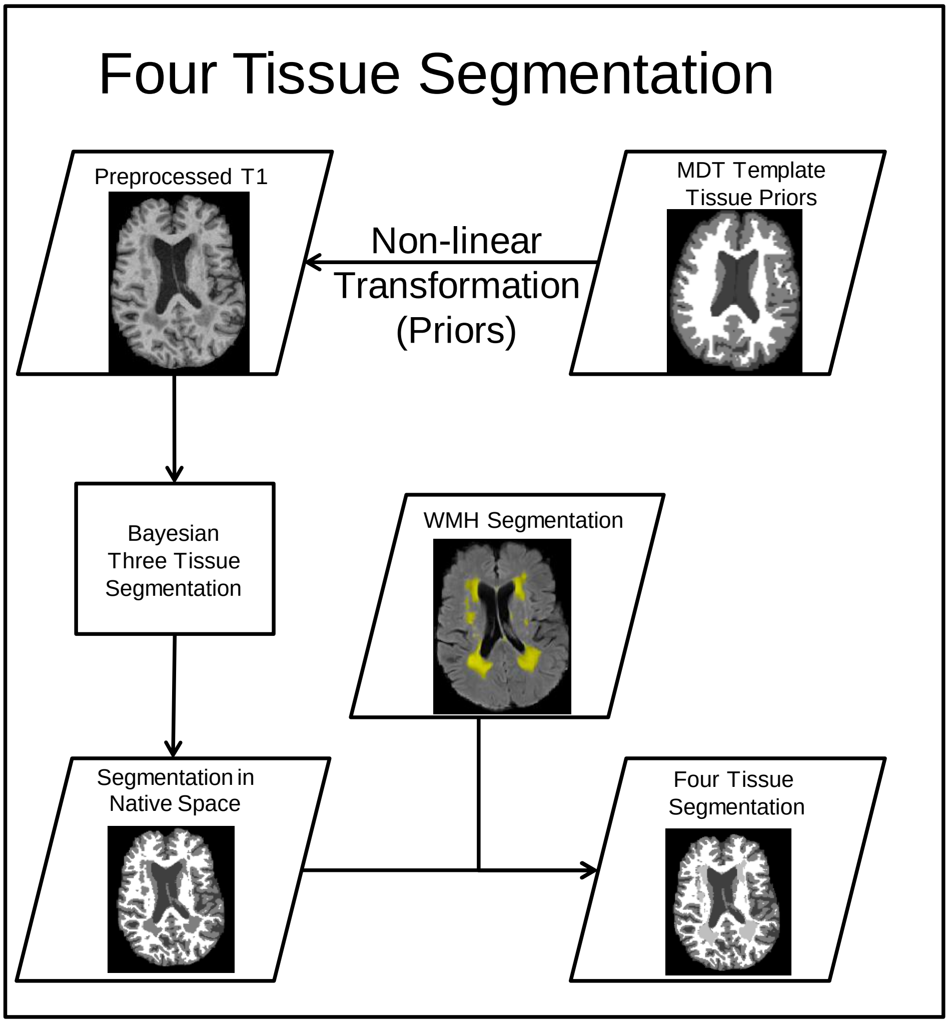

Supplementary Figure 2. Three and Four Tissue Segmentation
